# Learning curve and functional outcomes after laser enucleation of the prostate for benign prostate hyperplasia according to surgeon’s caseload

**DOI:** 10.1007/s00345-022-04177-y

**Published:** 2022-10-26

**Authors:** M. Kosiba, B. Hoeh, M. N. Welte, M. J. Krimphove, K. Vitucci, N. Lindemann, J. Schröder, L. Jost, F. E. Schmidt, A. von Hollen, L. A. Kluth, P. Mandel, F. C. Roos, F. K. H. Chun, A. Becker

**Affiliations:** 1 Department of Urology, University Hospital Frankfurt, Goethe University Frankfurt Am Ain, Theodor-Stern-Kai 7, 60590 Frankfurt am Main, Germany; 2 Cancer Prognostics and Health Outcomes Unit, Division of Urology, University of Montréal Health Center, Montréal, QC Canada

**Keywords:** HoLEP, SoLEP, Holmium:yag laser, Thulium fiber laser, Clavien−Dindo complications, Safety, Efficacy, Surgical education, Mentoring program

## Abstract

**Purpose:**

To evaluate the impact of surgical caseload on safety, efficacy, and functional outcomes of laser enucleation of the prostate (LEP) applying a structured mentoring program.

**Methods:**

Patient characteristics, perioperative data, and functional outcomes were analyzed descriptively. Linear and logistic regression models analyzed the effect of caseload on complications, functional outcomes and operative speed. Within the structured mentoring program a senior surgeon was present for the first 24 procedures completely, for partial steps in procedures 25–49, and as needed thereafter.

**Results:**

A total of 677 patients from our prospective institutional database (2017–2022) were included for analysis. Of these, 84 (12%), 75 (11%), 82 (12%), 106 (16%), and 330 patients (49%) were operated by surgeons at (A) < 25, (B) 25–49, (C) 50–99, (D) 100–199, and (E) ≥ 200 procedures. Preoperative characteristics were balanced (all *p > *0.05) except for prostate volume, which increased with caseload.

There was no significant difference in change of IPSS, Quality of life, ICIQ, pad usage, peak urine flow, residual urine, and major complications (Group A: 8.3 to E: 7.6%, *p = *0.2) depending on the caseload. Caseload was not associated (Odds ratio: 0.7–1.4, *p > *0.2) with major complications in the multivariable logistic regression model. Only operating time was significantly shorter with increasing caseload in the multivariable analysis (111–55 min, beta 23.9–62.9, *p < *0.001).

**Conclusion:**

With a structured mentoring program, the safety and efficacy of LEP can be ensured even during the learning curve with very good outcome quality. Only the operating time decreases significantly with increasing experience of the surgeon.

**Supplementary Information:**

The online version contains supplementary material available at 10.1007/s00345-022-04177-y.

## Introduction

Laser enucleation of the prostate (LEP) has proven to be a safe and effective minimally invasive surgical treatment for bladder outlet obstruction (BOO) due to benign prostate hyperplasia (BPH) or prostate cancer (PCa) [1, 2]. Unlike transurethral resection of the prostate (TURP), it can be performed regardless of size and with less morbidity as compared to TUR-P and open simple prostatectomy [3, 4], even in patients requiring anticoagulation [5]. Although multiple enucleation techniques, using various energy sources, have been described over the past few years [6, 7], HoLEP remains the only size-independent option validated in randomized controlled trials [8, 9] and supported by level-1 evidence [10, 11]. As such HoLEP is recommended by European Association of Urology (EAU) and American Urology Association (AUA) guidelines [12, 13] and has become the gold standard for the surgical treatment of lower urinary tract symptoms due to BOO [11, 14, 15]. Despite its scientific status, diffusion into clinical practice is limited and classical TURP remains the predominant surgical BOO procedure by far [16]. An allegedly flat learning curve is regarded as main barrier against widespread clinical acceptance [17–22]. However, more recently, a growing number of mentoring programs have been introduced, aiming to accelerate the learning curve [9, 22]. Especially one recent review [9] suggested that around 50 procedures might be sufficient to achieve a stable outcome, which could be reduced to about 25 in the case of structured training. We herein evaluated the learning curve of four novel LEP surgeons with regard to functional outcomes and complications. We postulated that applying a structured LEP mentoring program, stable functional outcomes and low complication rates can be achieved independent of the surgical caseload.

## Materials and methods

### Study population

**Fig. 1:**
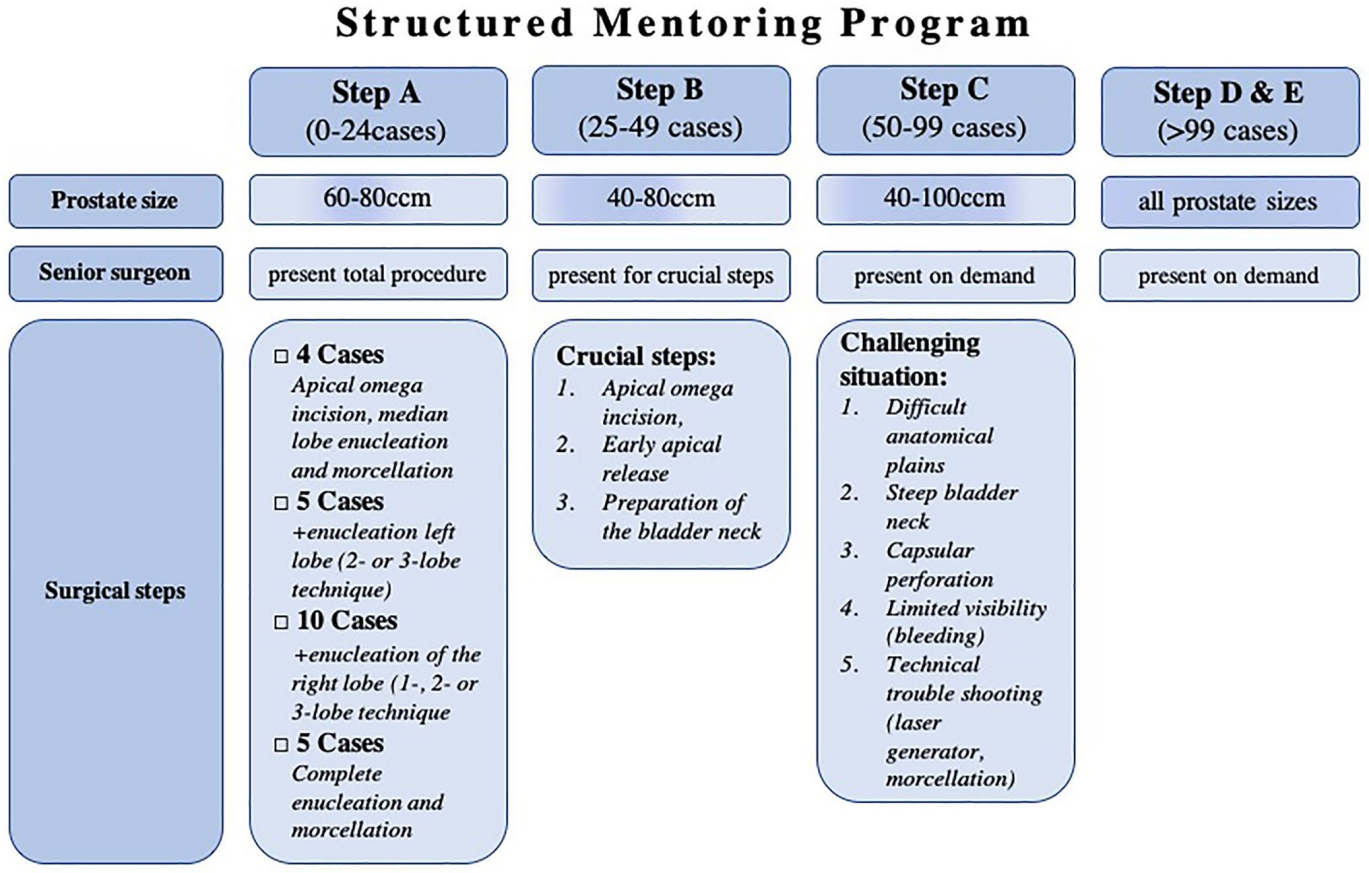
Structured mentoring program for laser enucleation of the prostate developed at University Hospital Frankfurt. In dependence of the novice surgeon's caseload, the appropriate prostate size, surgical steps and presence of a senior surgeon are determined. The mentoring program starts after observation of 10 full procedures conducted by expert surgeons (OR observation or video)

Within our prospectively maintained, institutional database, all consecutive patients who underwent LEP for BPH between November 2017 and January 2022 were identified. Patients with preoperative urethral strictures or previously known PCa were excluded (*n = *77). Procedures were performed by two senior LEP experts with experience of > 100 procedures (A.B., F.K.H.C.), as well as by four additional surgeons who learned LEP progressively using the structured mentoring program. A senior surgeon was present for the first 24 procedures completely, for partial steps in procedures 25–49, and as a second surgeon when needed after the 50th procedure. Patients were retrospectively stratified into five groups (A: 1–24, B: 25–49, C: 50–99, D: 100–199, E: ≥ 200 cases) depending on the operating surgeon’s caseload. Our mentoring program starts after observation of 10 procedures conducted by expert surgeons (OR observation or video) and is based on the curriculum displayed in Fig. 1.

### Surgical procedure

All surgical procedures were performed with the Olympus, OES Pro Laser Resectoscope, a high power (120 W) holmium laser generator (MOSES Pulse 120H, Lumenis) with a 550 nm laser fiber (Lumenis, Slim Line), or the Soltive™ SuperPulsed laser generator (Olympus) with a 550 nm laser fiber. For morcellation the PIRANHA morcellator by Richard Wolf or the Versacut morcellator by Lumenis was used.

### Follow-up

Standardized questionnaires (International Prostate Symptoms Score (IPSS), Quality of life (Qol), International Consultation on Incontinence Questionnaire (ICIQ), and pad usage) were assessed preoperatively and at 4 weeks, 3 months, and then yearly and delta (Δ) was calculated by subtracting postoperative from preoperative values. A pad usage of more than one security pad was defined as incontinent. Before surgery and at dismission, peak urine flow and residual urine was registered. Postoperative complications were recorded according to the Clavien−Dindo (CLD) classification system using a graduation from CLD I to CLD V [23]. Major complications were defined as CLD equal or greater IIIb [24]. Intraoperative complications were defined as any complication occurring during the procedure as f.ex capsular perforation or bladder injury.

### Statistical analysis

Descriptive statistics included frequencies and proportions for categorical variables. Means, medians, and interquartile-ranges (IR) were reported for continuously coded variables. The Chi-square test was used to assess the statistical significance in proportions’ differences. The *t* test and Kruskal−Wallis test examined the statistical significance of means’ and medians’ differences. Multivariable linear and logistic regression models were fit to analyze the effect of caseload on complications, functional outcomes, and OR speed. Covariates were age, prostate size, incidental PCa, American Society of Anesthesiologists (ASA) status and intraoperative complications.

In all statistical analyses, R software environment for statistical computing and graphics (version 3.6.1) was used. All tests were two-sided with a level of significance set at *p < *0.05. Ethical approval was obtained from the local ethics committee at the University Hospital Frankfurt. All included patients gave informed written consent.

## Results

In our institutional database, we identified 677 eligible patients who underwent LEP at the University Hospital Frankfurt from November 2017 to January 2022. Of these, 84 (12%), 75 (11%), 82 (12%), 106 (16%), and 330 patients (49%) were operated by surgeons at A: < 25, B: 25–49, C: 50–99, D: 100–199, and E: ≥ 200 procedures.

### Descriptive characteristics of the study population

Preoperative characteristics were balanced (Table 1) except for prostate volume, which increased significantly with increasing caseload (prostate volume in transrectal ultrasound (TRUS) 67 (group A) to 80ccm (group E), *p = *0.009). Overall 215 (35%) patients had an indwelling catheter before the surgery and 344 (74%) were continent, defined as usage of equal or less than one pad or ICIQ ≤ 4 ( the lowest amount over 0 in each ICIQ question). ASA score was predominantly low (452 patients (67%) had ASA I or II. After surgery an irrigation foley catheter was left for median 2 days, without significant differences among caseload groups. Operating room (OR) time was significantly reduced with increasing caseload, ranging from 111 min [Interquartile range (IQR): 91, 140 min] in group A to 55 min in group E (IQR 43, 76 min) (*p < *0.001), while enucleated prostate volume increased from 54 g in group A to 58 g in group E, *p = *0.03.

**Table 1:** Pre- and perioperative characteristics of 677 patients with laser enucleation of the prostate from the University Hospital Frankfurt between 11/2017 and 01/2022, stratified according to surgeon’s caseload

	*N*	Overall, *N = *677^a^	Caseload < 25, *N = *84 (12%)^a^	Caseload 25–49, *N = *75 (11%)^a^	Caseload 50–99, *N = *82 (12%)^1^	Caseload 100–199, *N = *106 (16%)^a^	Caseload > = 200, *N = *330 (49%)^a^	*p* value^b^
Age [years]	676	69 (63, 76)	69 (64, 76)	68 (62, 75)	70 (64, 76)	70 (64, 76)	69 (64, 75)	0.7
PSA [ng/ml]	567	4.3 (2.4, 8.1)	4.2 (2.2, 7.3)	4.3 (2.6, 7.2)	4.7 (2.5, 8.6)	5.3 (3.4, 10.4)	4.1 (2.2, 7.8)	0.2
TRUS [ccm]	670	79 (55, 101)	67 (50, 90)	66 (54, 86)	82 (51, 100)	80 (62, 110)	80 (55, 109)	**0.009**
OR time [min]	675	73 (51, 107)	111 (91, 140)	108 (72, 128)	89 (66, 133)	80 (60, 100)	55 (43, 76)	** < 0.001**
Enucleation volume [g]	673	55 (30, 82)	54 (26, 70)	45 (26, 75)	51 (27, 68)	60 (38, 90)	58 (33, 85)	**0.03**
Catheter time [days]	652	2.0 (2.0, 2.0)	2.0 (2.0, 2.0)	2.0 (2.0, 2.0)	2.0 (2.0, 2.0)	2.0 (2.0, 2.0)	2.0 (2.0, 2.0)	0.3
ASA status	676							0.3
I/II		452 (67%)	53 (63%)	48 (65%)	49 (60%)	68 (64%)	234 (71%)	
III/IV		224 (33%)	31 (37%)	26 (35%)	33 (40%)	38 (36%)	96 (29%)	
preOP transurethral catheter (n,%)	614	215 (35%)	35 (44%)	28 (42%)	28 (39%)	38 (37%)	86 (29%)	0.07

Bold values indicate the *p*-values represent significant values (*p* < 0.05)

*PSA* prostate-specific antigen; *TRUS* prostate volume in transrectal ultrasound; *OR* operating room; *ASA* American Society of Anesthesiologists Physical Status Classification System

^a^Median (Interquartile range IQR); *n* (%)

^b^Kruskal−Wallis rank sum test; Pearson’s Chi-square test

### Postoperative outcomes

There was no significant difference between caseload groups with regard to follow up measures (Table 2). International Prostate Symptoms Score (IPSS) was reduced by median 11 (IQR 5–17) points as compared to preoperatively. The quality of life (Qol) was improved by median 3 (IQR 1–4) points. The median ICIQ score or pad consumption did not change significantly as compared to preoperatively. Peak urine flow was increased by median 9 ml/s (IQR 3–16 ml/s) and residual urine was reduced by median 60 ml (IQR 15-150 ml). Major complications were not recorded significantly more often at the beginning of the learning curve than in more experienced levels (group A: 8.3% vs group E: 7.6%, *p = *0.2). Indwelling transurethral catheter was reduced from preoperative overall 210 (35%) patients to 25 (3.8%) at dismission, without significant differences according to caseload. Finally, 443 patients (95% (A: 91% to E: 97%, *p = *0.2) were continent at last follow up (Table 2, rates over time shown in Supplemental Fig. 3).

**Table 2:** ostoperative Follow up characteristics of 667 patients with laser enucleation of the prostate from the University Hospital Frankfurt between 11/2017 and 01/2022, stratified according to surgeon’s caseload

	*N*	Overall, *N = *677^a^	Caseload < 25, *N = *84 (12%)^a^	Caseload 25–49, *N = *75 (11%)^a^	Caseload 50–99, *N = *82 (12%)^a^	Caseload 100–199, *N = *106 (16%)^a^	Caseload > = 200, *N = *330 (49%)^a^	*p* value^b^
Δ IPSS	224	11 (5, 17)	8 (3, 14)	13 (3, 17)	14 (6, 18)	14 (8, 16)	11 (6, 17)	0.6
Δ Qol	377	3.0 (4.0, 1.0)	2.0 (4.0, 1.0)	2.0 (4.0, 0.0)	3.0 (4.0, 1.0)	3.0 (4.0, 2.0)	3.0 (4.0, 1.0)	0.3
Δ ICIQ	341	0.0 (-2.0, 4.0)	0.0 (-2.5, 5.0)	0.0 (-0.5, 4.5)	0.0 (-5.8, 3.8)	0.0 (0.0, 2.0)	0.0 (-2.0, 4.0)	0.9
Δ pads	160	0.0 (0.0, 1.0)	0.0 (-0.75, 1.0)	0.0 (0.0, 1.0)	0.0 (0.0,1.0)	0.0 (-1.50, 0.0)	0.0 (0.0, 1.0)	0.2
Δ *Q*max	257	9 (3, 16)	10 (4, 15)	12 (3, 20)	9 (5, 14)	10 (2, 16)	8 (4, 14)	0.8
Δ Post void residual urine	344	60 (15,150)	56 (1, 128)	100 (42, 226)	80 (18, 150)	72 (14, 200)	50 (15, 138)	0.5
30 day-complications	667							0.2
None		486 (72%)	58 (69%)	49 (65%)	56 (68%)	78 (74%)	245 (74%)	
Minor (Clavien Dindo ≤ 3a)		142 (21%)	19 (23%)	24 (32%)	17 (21%)	22 (21%)	60 (18%)	
Major (Clavien Dindo ≥ 3b)		49 (7.2%)	7 (8.3%)	2 (2.7%)	9 (11%)	6 (5.7%)	25 (7.6%)	
Post OP transurethral catheter	664	25 (3.8%)	5 (6.1%)	2 (2.7%)	4 (4.9%)	5 (4.7%)	9 (2.8%)	0.5
Continence^c^	464							0.2
Post OP continent		443 (95%)	48 (91%)	50 (94%)	42 (91%)	72 (97%)	231 (97%)	
De novo incontinent		18 (3.9%)	4 (7.5%)	2 (3.8%)	4 (8.7%)	2 (2.7%)	6 (2.5%)	
Preoperatively incontinent		3 (0.6%)	1 (1.9%)	1 (1.9%)	0 (0%)	0 (0%)	1 (0.4%)	

*IPSS* International Prostate Symptoms Score; *Qol* quality of life; *ICIQ* International Consultation on Incontinence Questionnaire; Qmax, peak urine flow

^a^Median (Interquartile range IQR); *n* (%)

^b^Kruskal–Wallis rank sum test; Pearson's Chi-square test; Fisher's exact test

^c^Defined as usage of 0–1 pads per day

### Multivariable linear regression

OR time and isolated enucleation time was univariably plotted according to surgeon’s caseload (Supplemental Fig. 2). A multivariable linear regression model was fitted to predict OR time according to surgeon’s caseload (Supplemental Table 3). Covariates consisted of TRUS prostate volume, age, ASA status, intraoperative complications and incidental prostate carcinoma. Caseload showed a highly significant and consistent decrease in OR time with every increment of experience (beta 62.86 to 23.92 min, all *p < *0.01). Intraoperative complications (beta 14.37 min) and TRUS prostate volume (beta 0.48) were significant predictors for increasing OR time (all *p < *0.01).

### Multivariable logistic regression

Multivariable logistic regression model was fitted to predict major complications according to surgeon’s caseload, adjusted for TRUS prostate volume, age, ASA status, and incidental prostate carcinoma (Supplemental Table 4). Caseload was not a significant predictor of complications, regardless of its increment. However, Prostate size (OR 1.01), and high ASA status (OR 2.29) were significant predictors for major complications (all *p < */ = 0.01). Diagnosis of incidental prostate carcinoma (OR 1.9) as predictor of major complications did not reach statistical significance (*p = *0.09).

## Discussion

We reported our institutional experience regarding safety and efficacy of LEP with regard to surgeon’s caseload. Our analyses yielded several important observations.

First, we reported a large series of nearly 700 patients, which is one of the biggest cohorts published concerning learning curve analyses [17–19]. Moreover, our report does not represent a single surgeon´s experience but pooled results from four beginner surgeons, which make our results very robust and less susceptible to individual abilities and, as such, more generalizable.

Second, we found a highly significant and consistent decrease in OR time depending on surgeons’ caseload. This finding is plausible and consistent with virtually all previous reports that also confirmed a more time efficient procedure with growing experience. Brunckhorst et al. [12] reported a retrospective analysis of 253 consecutive cases of a single HoLEP surgeon. Consistent with our results, they found that enucleation speed was significantly increasing throughout the beginning of the learning curve, however, they found a plateau after 50–60 cases which is in contrast to our results, where we saw ongoing improvements in OR time until > 200 cases.

Third, we found excellent and stable functional outcomes independent of the surgeon’s caseload. This is in contrast to most of the other studies as highlighted in a systematic review by Kampantais et al. [9], which included 24 studies with a total of 5173 patients. They found ongoing improvements in functional outcomes until a plateau was reached after approximating 25–50 cases. This is in contrast to our findings, where we found stable functional outcomes for novices trained within our structured mentoring program independent of their caseload. However, the authors also concluded that a structured mentorship program would aid for faster progress, which might explain our better results.

Fourth, we evaluated major complication rates according to caseload and found no significant association between caseload and occurrence of major complications. Interestingly, the majority of publications report on functional outcomes or OR time. Only a minority of studies also reported on complications, when assessing the learning curve. This is worrisome as a low complication rate should be the utmost goal for every intervention, especially for a surgery, which is conducted for a benign disease. Rosenhammer et al. [25] found that ASA status and prostate cancer were independent predictors for grade ≥ 2 complications but not the caseload, which is consistent with our results, where we also found ASA status and prostate volume, but not caseload, as significant predictor of major complications. Moreover, Westhofen et al. [22] found the overall incidence of treatment-related adverse events was significantly higher without a training program. Conversely, Lerner et al. [19] showed a strong correlation between complication rates and learning curve in their study, especially when time between consecutive cases was more than 5 weeks. We did not evaluate the time in between the consecutive cases of the surgeons in our analysis, so we cannot compare these results. However, it appears logical especially without a structured mentoring program that already learned skills will diminish when time is too long in between two cases. Similarly, the institutional caseload seems important in this regard, as beside personal frequency of interventions, a high level of competence of the team in dealing with the intervention ensures high quality surgical outcomes [26].

Fifth, we applied Trifecta and Pentafecta criteria as defined by Robert et al. [21] to our results. These criteria of a perfect procedure consist of the combination of complete enucleation and morcellation within less than 90 min and without any conversion to standard TURP (Trifecta). In addition, information on complications or occurrence of stress urinary incontinence in 3 months after the operation are added for the Pentafecta criteria. Applying these criteria to our results, we achieved the Trifecta criteria mainly by reaching the expected OR time of less than 90 min after more than 50 cases, as we never used conversion to TUR-P. Similarly, the Pentafecta criteria were reached at 50 cases due to OR time because of consistently low rates of complications and urinary incontinence. This is comparable to the report by Peyronnet et al. [20] who met the criteria in four consecutive patients after the 40th procedure in the HoLEP group.

Taken together, we reported on a large series of nearly 700 patients with pooled results from four beginner surgeons, which make our results very robust and pertinent. We found a highly statistically significant improvement in OR time according to surgeon’s caseload. This is in accordance to virtually all other reports. However, we found stable functional outcomes and stable low complications rates, independent from the surgeon’s caseload. This is in contrast to many other studies that report an improvement of these variables according to the surgeon’s caseload. Apparently, a structured mentoring program over the first 50 cases, which was applied in our institution can help overcome the learning curve.

Several limitations apply to our study. Although the database is designed prospectively, the analysis of the learning curve is retrospective and thus selection biases may occur. Low number of cases in each caseload group limited depth of subgroup analyses. Furthermore, our prospective database may be influenced by a negative selection bias regarding the admittance of patients with a particularly high perioperative risk to our tertiary care university center. Nevertheless, we included all LEP patients since its implementation at our institution to allow most comprehensive analyses.

## Conclusion

With a structured mentoring program, the safety and efficacy of LEP can be ensured even during the learning curve with excellent functional outcomes. Only the OR time decreases significantly with increasing experience of the surgeon.

## Supplementary Information

Below is the link to the electronic supplementary material.

Supplementary file1 (DOCX 15 kb)

Supplementary file2 (DOCX 15 kb)

Supplementary file3 (PDF 92 kb)

Supplementary file4 (PDF 32 kb)

## Data Availability

The data are available for requests.
