# Supplementary material for: Learning curve and functional outcomes after laser enucleation of the prostate for benign prostate hyperplasia according to surgeon’s caseload
Source: World J Urol. 2022 Oct 26;40(12):3007–13. doi: 10.1007/s00345-022-04177-y (PMC9712403; doi:10.1007/s00345-022-04177-y)
Supplement: Supplementary file 1 — Supplementary file1 (DOCX 15 kb) [file 345_2022_4177_MOESM1_ESM.docx]

**Table 3.**

|  | Beta | 95% CI1 | p-value |
| --- | --- | --- | --- |
| **caseload** |  |  |  |
| ≥200 | Ref. | — |  |
| <25 | 62.86 | 55.14, 70.59 | **<0.001** |
| 25-49 | 52.34 | 44.41, 60.26 | **<0.001** |
| 50-99 | 42.01 | 34.34, 49.68 | **<0.001** |
| 100-199 | 23.92 | 17.07, 30.76 | **<0.001** |
| **Prostate carcinoma** | -4.96 | -11.70, 1.78 | 0.15 |
| **Age** | 0.26 | -0.04, 0.57 | 0.09 |
| **TRUS** | 0.48 | 0.42, 0.54 | **<0.001** |
| **Intraop.** **complications** | 14.37 | 7.22, 21.53 | **<0.001** |
| **ASA status** |  |  |  |
| I/II | — | — |  |
| III/IV | 4.75 | -0.52, 10.01 | 0.08 |
| *1CI = Confidence Interval* | | | |

Multivariable linear regression model predicting OR time according to surgeon’s caseload, adjusted for size of prostate, age, ASA status, intraoperative complications and incidental of prostate carcinoma.

*Abbreviations: TRUS- prostate volume in transrectal ultra sound, OR- operating room, ASA- American Society of Anesthesiologists Physical Status Classification System*
