# Supplementary material for: Learning curve and functional outcomes after laser enucleation of the prostate for benign prostate hyperplasia according to surgeon’s caseload
Source: World J Urol. 2022 Oct 26;40(12):3007–13. doi: 10.1007/s00345-022-04177-y (PMC9712403; doi:10.1007/s00345-022-04177-y)
Supplement: Supplementary file 2 — Supplementary file2 (DOCX 15 kb) [file 345_2022_4177_MOESM2_ESM.docx]

**Table 4.**

|  | OR1 | 95% CI1 | p-value |
| --- | --- | --- | --- |
| **Caseload** |  |  |  |
| ≥200 | Ref. | — |  |
| <25 | 1.45 | 0.53, 3.54 | 0.44 |
| 25-49 | 0.42 | 0.07, 1.52 | 0.26 |
| 50-99 | 1.62 | 0.67, 3.66 | 0.26 |
| 100-199 | 0.74 | 0.26, 1.81 | 0.54 |
| **TRUS** | 1.01 | 1.01, 1.02 | **<0.001** |
| Age | 1.04 | 0.99, 1.08 | 0.08 |
| **Prostate carcinoma** | 1.91 | 0.87, 3.98 | 0.09 |
| **ASA status** |  |  |  |
| I/II | Ref. | — |  |
| III/IV | 2.29 | 1.22,4.36 | **0.01** |
| *1OR = Odds Ratio, CI = Confidence Interval* | | | |

Multivariable logistic regression model predicting major complications according to surgeon’s caseload, adjusted for size of prostate, age, ASA status and incidental prostate carcinoma.

*Abbreviations: TRUS- prostate volume in transrectal ultra sound, OR- operating room, ASA- American Society of Anesthesiologists Physical Status Classification System*
