# Supplementary material for: Learning curve and functional outcomes after laser enucleation of the prostate for benign prostate hyperplasia according to surgeon’s caseload
Source: World J Urol. 2022 Oct 26;40(12):3007–13. doi: 10.1007/s00345-022-04177-y (PMC9712403; doi:10.1007/s00345-022-04177-y)

Figure 2.
Enucleation time and operating time of 677 patients with laser enucleation of the prostate from the University Hospital Frankfurt between 11/2017 and 01/2022, stratified according to surgeon's caseload.

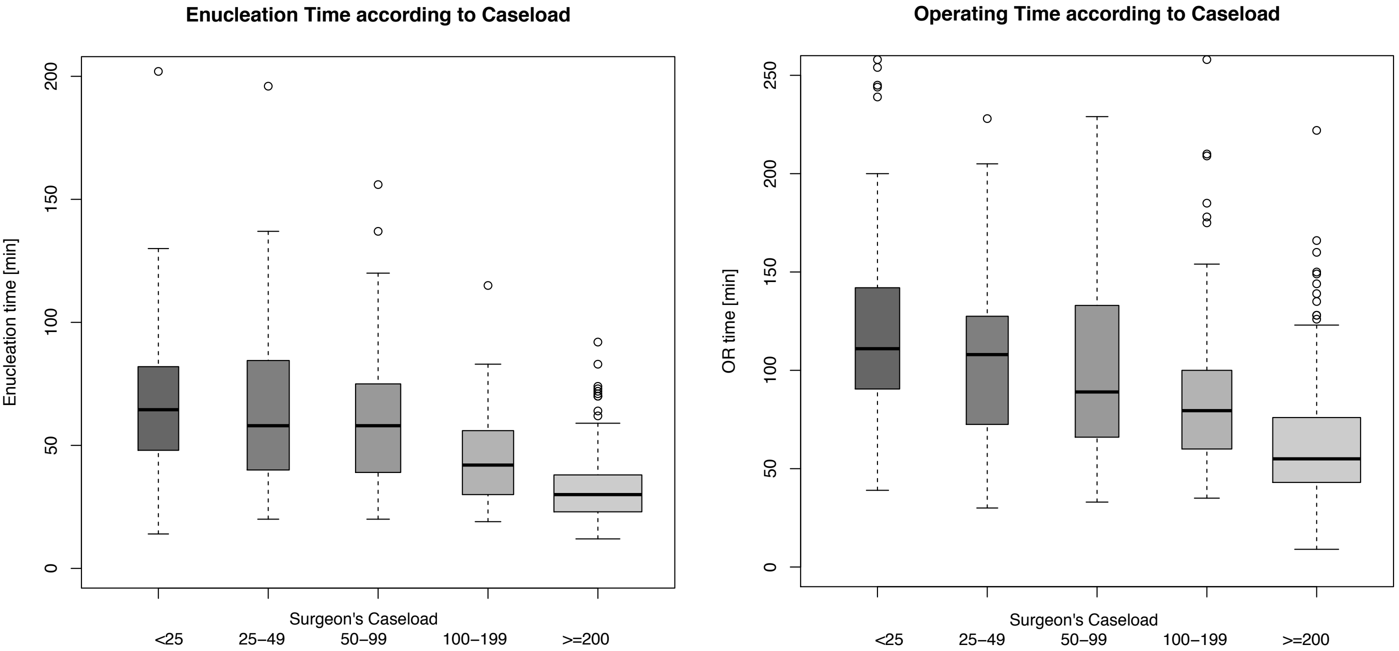

Supplement: Supplementary file 3 — Supplementary file3 (PDF 92 kb) [file 345_2022_4177_MOESM3_ESM.pdf]
