# Supplementary figures and images for: Learning curve and functional outcomes after laser enucleation of the prostate for benign prostate hyperplasia according to surgeon’s caseload
Source: World J Urol. 2022 Oct 26;40(12):3007–13. doi: 10.1007/s00345-022-04177-y (PMC9712403; doi:10.1007/s00345-022-04177-y)

Mean ICIQ values over time according to caseload group

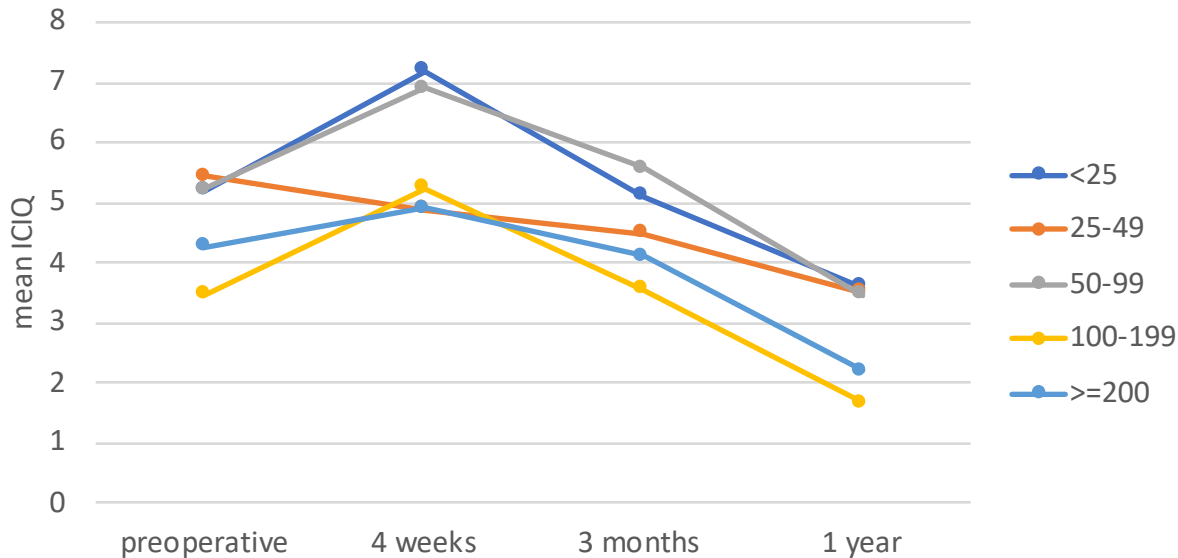

Supplement: Supplementary file 4 — Supplementary file4 (PDF 32 kb) [file 345_2022_4177_MOESM4_ESM.pdf]
